# Phylogenetic Distribution of Fungal Sterols

**DOI:** 10.1371/journal.pone.0010899

**Published:** 2010-05-28

**Authors:** John D. Weete, Maritza Abril, Meredith Blackwell

**Affiliations:** 1 Auburn Research and Technology Foundation, Auburn, Alabama, United States of America; 2 Department of Biological Sciences, Louisiana State University, Baton Rouge, Louisiana, United States of America; University College Dublin, Ireland

## Abstract

**Background:**

Ergosterol has been considered the “fungal sterol” for almost 125 years; however, additional sterol data superimposed on a recent molecular phylogeny of kingdom Fungi reveals a different and more complex situation.

**Methodology/Principal Findings:**

The interpretation of sterol distribution data in a modern phylogenetic context indicates that there is a clear trend from cholesterol and other Δ^5^ sterols in the earliest diverging fungal species to ergosterol in later diverging fungi. There are, however, deviations from this pattern in certain clades. Sterols of the diverse zoosporic and zygosporic forms exhibit structural diversity with cholesterol and 24-ethyl -Δ^5^ sterols in zoosporic taxa, and 24-methyl sterols in zygosporic fungi. For example, each of the three monophyletic lineages of zygosporic fungi has distinctive major sterols, ergosterol in Mucorales, 22-dihydroergosterol in Dimargaritales, Harpellales, and Kickxellales (DHK clade), and 24-methyl cholesterol in Entomophthorales. Other departures from ergosterol as the dominant sterol include: 24-ethyl cholesterol in Glomeromycota, 24-ethyl cholest-7-enol and 24-ethyl-cholesta-7,24(28)-dienol in rust fungi, brassicasterol in Taphrinales and hypogeous pezizalean species, and cholesterol in *Pneumocystis*.

**Conclusions/Significance:**

Five dominant end products of sterol biosynthesis (cholesterol, ergosterol, 24-methyl cholesterol, 24-ethyl cholesterol, brassicasterol), and intermediates in the formation of 24-ethyl cholesterol, are major sterols in 175 species of Fungi. Although most fungi in the most speciose clades have ergosterol as a major sterol, sterols are more varied than currently understood, and their distribution supports certain clades of Fungi in current fungal phylogenies. In addition to the intellectual importance of understanding evolution of sterol synthesis in fungi, there is practical importance because certain antifungal drugs (e.g., azoles) target reactions in the synthesis of ergosterol. These findings also invalidate use of ergosterol as an indicator of biomass of certain fungal taxa (e.g., Glomeromycota). Data from this study are available from the Assembling the Fungal Tree of Life (AFTOL) Structural and Biochemical Database: http://aftol.umn.edu.

## Introduction

**Figure 1:**
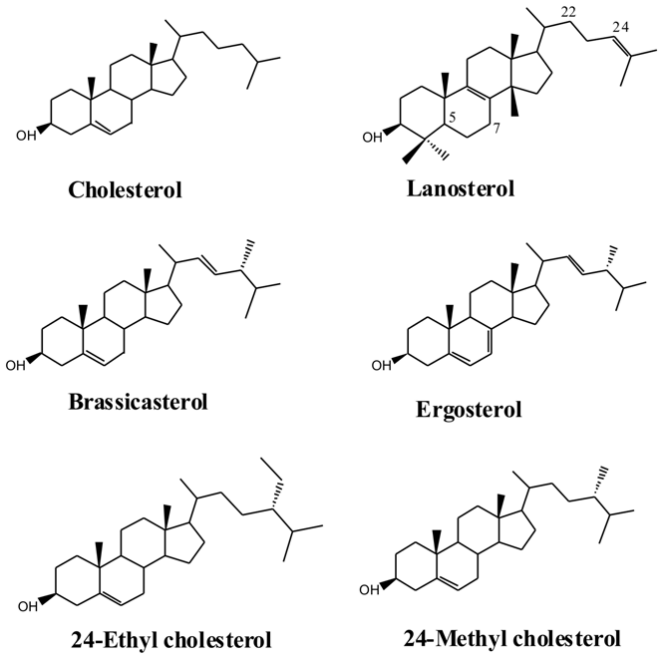
Structures of main fungal sterols. (A) Cholesterol (B) Lanosterol (C) Brassicasterol (D) Ergosterol (E) 24-Ethyl cholesterol and (F) 24-Methyl cholesterol.

**Figure 2:**
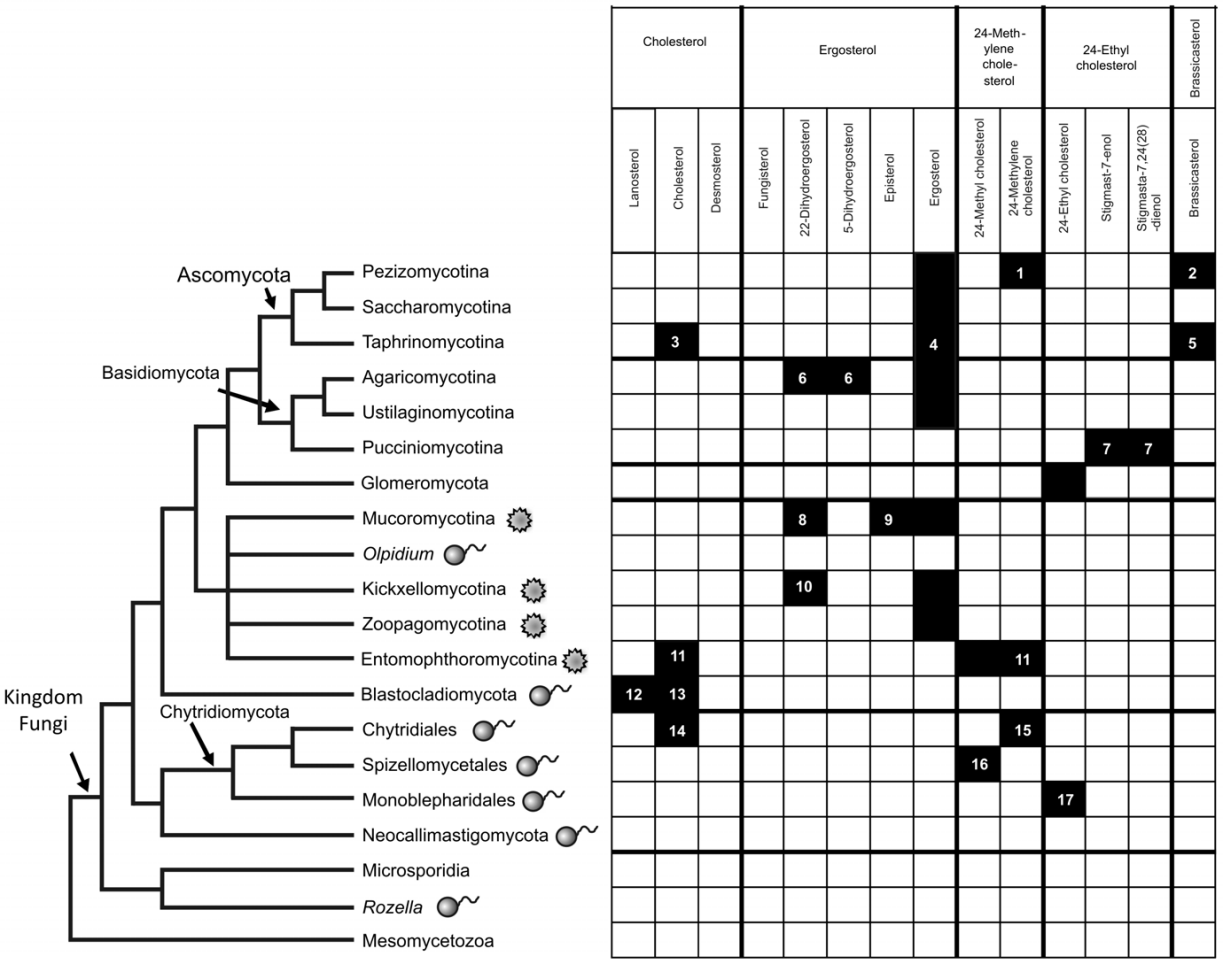
Fungal phylogenetic tree based on James et al. (2006), White et al. (2006), and Hibbett et al. (2007), and major sterols associated with each taxon. The zygosporic fungi are indicated by flagellated cell diagrams and zygosporic fungi, by zygospore diagrams. Filled squares indicate the predominant sterol present within a terminal taxon (usually a phylum). Distinctive sterols are present in some clades within phyla: 1 Erysiphales; 2 *Tuber* and *Terfezia* also contain some ergosterol; 3 *Pneumocystis*; 4 *Schizosaccharomyces*; 5 *Taphrina* and *Protomyces*; 6 several core polypores; 7 Pucciniales; 8 one member of Mucorales reported to contain 22-dihydroergosterol; 9 several members of Mucorales contain episterol; 10 Kickxellales; 11 Most members of Entomophthorales contain 24-methyl cholesterol although several are known to have cholesterol or 24-methylene cholesterol or mixtures of 24-methyl cholesterol and 24-methylene cholesterol; 12 *Catenaria anguillulae*; 13 *Allomyces macrogynus*; 14 most species of Chytridiales tested; 15 *Rhizophydium sphaerotheca*; 16 only *Spizellomyces punctatum* sampled; 17 only *Monoblepharella* sp. sampled. Mesomycetozoa (Mendoza et al. 2002) is considered to be the sister group to fungi, and this group is poorly sampled. Note that many clades are undersampled or completely unsampled (see text for details).

Sterols are required for fungal growth, a fact that has been exploited in the development of antifungal pesticides widely used in agriculture and antimycotics used to control fungal diseases of humans and animals. Since 24R-methyl-cholesta-5,7,22(E)-trienol (ergosterol; C_28_ Δ^5,7,22^) (Figure 1) was discovered over 100 years ago in the plant pathogenic ergot fungus *Claviceps purpurea* [1], it has been considered to be the “fungal sterol.” Ergosterol is not present in all fungi, and the misconception came about because most of the first fungi analyzed for sterols were among later diverging species (Ascomycota and Basidiomycota) in which ergosterol is dominant [2]. Ergosterol became so established as the sole fungal sterol that it has been used as a marker to estimate fungal biomass in plants and soils [3]–[5]. However, greatly improved information on the distribution of sterols (Figures 1 and 2; Table S1) across the kingdom Fungi since the mid-1970s [2], [6]–[8] revealed that the situation is not so simple.

Our understanding of fungal phylogeny also has advanced dramatically over the past 20 years by analyses of multiple DNA loci and increased taxon sampling. The analyses support a monophyletic group of organisms with more diversity in the early diverging lineages than previously recognized [9]–[11] (Figure 2).

**Figure 3:**
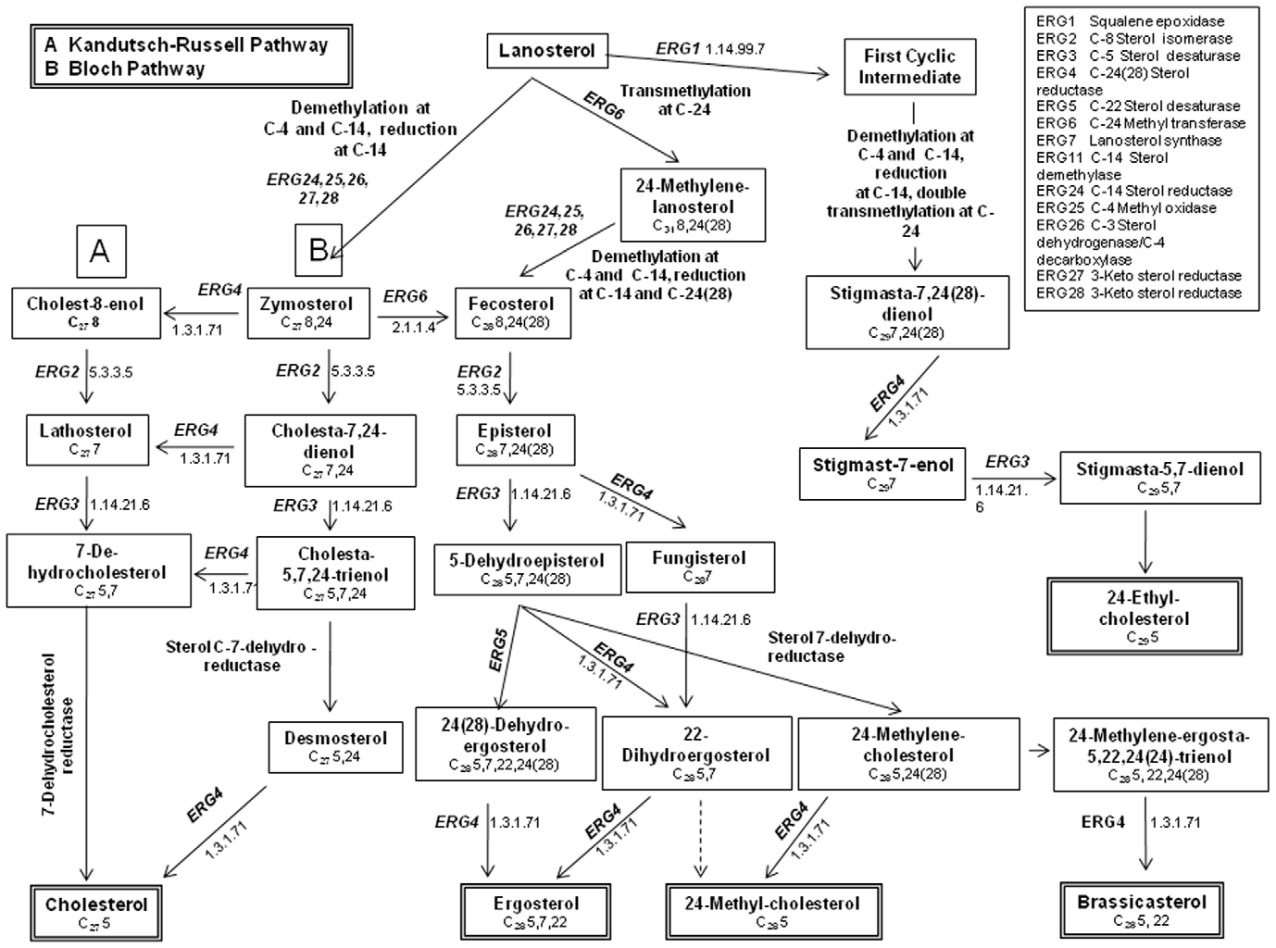
Diagram of the five predominant end products of sterol biosynthesis in fungi. Cholesterol, ergosterol (the primary sterol in many groups of fungi), 24-methyl cholesterol, 24-ethyl cholesterol, and brassicasterol and intermediates in the formation of 24-ethyl cholesterol, as major sterols reported in 175 fungal species across the kingdom Fungi.

As in cholesterol biosynthesis in animals, lanosterol (Figures 1 and 3), is the first cyclic intermediate in the formation of sterols in fungi. Most fungal sterols are distinguished by the methylation of lanosterol at C-24, and thereafter follow the same series of demethylations at C-4 and C-14 and double bond transformations as in cholest-5-enol (cholesterol) biosynthesis that lead to C_28_ sterols common in most fungi.

Multiple pathways leading to the formation of the C_28_ sterol ergosterol vary according to the sequence of double bond transformations. In some taxa, the pathways to ergosterol are incomplete and in some cases result in the formation of other end-products (i.e., not converted to ergosterol). A second methylation resulting in a 24-ethylidene, which is reduced to 24-ethyl, leads to C_29_ sterols in some taxa. The pathways for the five major sterol end-products, and C_29_ sterols, are summarized in Figure 3.

The distribution of sterols in the kingdom Fungi from the early diverging lineages, including zoosporic and zygosporic forms, to the most advanced taxa of the Ascomycota and Basidiomycota is discussed in light of recent phylogenetic analyses [9]–[11] (Figure 2).

## Methods

Sterols in fungi typically exist as a mixture of several sterols with one that is dominant, i.e., representing over 50% of the total sterol composition. The dominant or major sterol is most often accompanied by other sterols that are typically intermediates in the synthesis of the major sterol. The discussion of the distribution of sterols herein is in the context of the major or dominant sterol that accumulates in a fungus (Figure 2). For clarification, the systematic name of a sterol is given on its first use followed by the common name in parentheses, and the common name is used thereafter. Structures of the major sterols discussed in this paper are shown in Figure 1. Sterol presence is summarized and mapped on a phylogenetic tree (Figure 2).

A variety of methods were used in the isolation and characterization of sterols discussed in this paper as applied by the authors of the literature cited herein. Where anomalies exist, identifications based on the most advanced instrumentation (GLC-MS; H^1^NMR) are given the most weight as opposed to less specific methods (absorption spectra, etc).

The NCBI database was searched for genes (Fig. 3) in the sterol synthesis pathway, but no unexpected or insightful results were revealed, largely because of absence of data on early diverging fungal lineages.

## Results

The most species diverse, later diverging phyla, Ascomycota and Basidiomycota, form a well-supported monophyletic group (Figure 2). These taxa constitute the vast majority of fungi with approximately 64,000 and 32,000 species [12], respectively, and most species sampled have ergosterol as the major sterol component (Table S1).

However, it is clear now that ergosterol is not universally distributed among fungi as once believed, but instead there appear to be at least five taxon-specific end-products of sterol biosynthesis that occur as dominant sterols in certain lineages, including some subclades of Ascomycota and Basidiomycota. For example, the rust fungi tend to have intermediates in 24-ethyl cholesterol biosynthesis as major sterols. A trend from cholesterol to intermediates in the ergosterol pathway to ergosterol is evident in some taxa. In many cases, specific sterols occur as distinctive major sterols in certain lineages (Figure 2; Table S1).

A search in GenBank of genes involved in sterol synthesis did not reveal any unexpected findings at the time this paper was written, but genomes of critical taxa were not yet available. The results from a number of new fungal genome initiatives, however, are expected within several years, and this report will serve as a basis for their investigation. Data from this study also are available from the Assembling the Fungal Tree of Life (AFTOL) Structural and Biochemical Database <http://aftol.umn.edu>.

### Zoosporic Fungi

The zoosporic fungi (chytrids) are now placed among three phyla (Chytridiomycota, Neocallistigomycota, and Blastocladiomycota). Other flagellated species, both endobiotic parasites (*Rozella* and *Olpidium*), occur outside of these phyla in independent lineages with the implication that flagella were lost on several occasions [9]. Relatively few zoosporic fungi have been tested for sterols, and these are all members of the Chytridiomycota and Blastocladiomycota (Figure 2; Table S1).

#### Chytridiomycota

Members of only three orders of the diverse Chytridiomycota have been analyzed for sterol composition (Figure 2; Table S1). Four species of Chytridiales have cholesterol as the major sterol, and one species contained 24-methyl cholesterol as the dominant sterol [13]. Only one species of the Spizellomycetales has been investigated and has 24-methyl cholesterol as the primary sterol. A single species of Monoblepharidales has 24-ethyl cholesterol as the major sterol. Although some of the few zoosporic fungi surveyed are known to produce 24-methyl sterols, they are missing double bonds at C-7 and C-22, unlike some of the ergosterol-containing zygomycetes in the Mucoromycotina lineage.

#### Blastocladiomycota

Two species of Blastocladiomycota have cholesterol as the major sterol, and one accumulates lanosterol [13] (Figure 2; Table S1).

### Zygosporic Fungi

The placement of zygosporic fungi (zygomycetes) is not well-resolved, and they have been maintained as separate subphyla rather than united in a single phylum [11] (Figure 2). There is evidence, however, to suggest that zygosporic fungi are distributed among three monophyletic lineages, each with its distinctive dominant sterol (Figure 2; Table S1). These taxa center on 1) Mucorales, 2) members associated with several insect and invertebrate animal groups (DHK clade), and 3) Entomophthorales [10]. Seven major sterols [ergosterol, 24-methyl-cholesta-7,24(28)-dienol (episterol), 24-methyl-cholesta-5,7,-dienol (22-dihydroergosterol), cholesta-5,24-dienol (desmosterol), 24-methyl-cholesta-5,24(28)-dienol (24-methylene cholesterol), 24-methyl cholesterol, and cholesterol] are distributed among the zygosporic orders, and some are phylogenetically informative (Figure 2; Table S1).

#### Mucoromycotina

This lineage of zygosporic fungi (Mucorales, Endogonales, and Mortierellales) appears to be a monophyletic group [10]. Ergosterol is the major sterol in a diverse sampling of Mucorales (Figure 2; Table S1) [7]. No species of the Endogonales has been analyzed for sterol composition.

Ergosterol is absent, however, from Mortierellales. The distribution of major sterols varies among Mortierellales depending on the species, but includes desmosterol, 24-methylene-cholesterol, and 24, 25-methylene-cholesterol or combinations of these sterols [8] (Figure 2; Table S1). Separation of the order from Mucorales was first suggested by these results and corresponds with findings of morphological and molecular studies [10].

#### Kickxellomycotina

*Dimargaritales, Harpellales, and Kickxellales (DHK Clade)*. The DHK clade of the zygosporic fungi includes Dimargaritales, Harpelles, and Kickxellales (DHK Clade, Figure 2). Most members of this clade accumulate ergosterol as their major sterol. The exceptions are several species of the Kickxellales that have 22-dihydroergosterol, an intermediate in ergosterol biosynthesis, as the major sterol, and contain no ergosterol [7] (Figure 2; Table S1). No member of the Harpellales has been analyzed for sterol composition.

#### Zoopagomycotina

The Zoopagomycotina may also belong in the DHK clade (see below), but we have maintained it in a separate lineage for now [11] (Figure 2; Table S1). In addition to molecular analyses, septal pore structure was used as early evidence to suggest a relationship among DHK clade members [14]–[16]. Only two species of the Zoopagales (*Syncephalis spherica* and *Zoophagus insidians*) have been investigated, and both are reported to have ergosterol as their major sterol [7], [17] (Figure 2; Table S1). It is interesting that *Z. insidians* was reported to be a pythiaceous oomycete, and the presence of ergosterol supports the placement of this species as a fungus rather than an oomycete. The Oomycetes in which pythiacious fungus-like organisms are placed do not produce sterols [2].

#### Entomophthorales

In the third lineage of zygosporic fungi, all of the *Conidiobolus* and *Entomophthora* species analyzed contained 24-methyl cholesterol as the major sterol (Figure 2; Table S1). The position of Basidiobolaceae, containing zygosporic insect associates previously considered to be members of Entomophthorales, remains unresolved. Like the Entomophthorales, the only species of the monotypic family analyzed is *Basidiobolus ranarum*, which has 24-methyl cholesterol as the major sterol [7] (Figure 2; Table S1).

### Glomeromycota

The Glomeromycota, arbuscular mycorrhizal fungi, are associated with the roots of many plants. More recently, 24-ethyl cholesterol (C_29_ Δ^5^) has been reported as the major sterol of four species of the phylum, and ergosterol has not been detected [18] (Figure 2; Table S1).

### Dikarya

Most of the Ascomycota and Basidiomycota have ergosterol as their major sterol, but there are several notable exceptions that distinguish certain clades within each phylum.

### Ascomycota

#### Taphrinomycotina

This ascomycete subphylum is varied in many of its morphological and biochemical characters, including the major sterol production. *Pneumocystis carinii* has been shown to synthesize cholesterol, and a broad sampling of species of *Taphrina* and *Protomyces* have 24-methyl-cholesta-5,22-dienol (brassicasterol) as their primary sterol. *Schizosaccharomyces pombe* produces ergosterol [19] (Figure 2; Table S1).

#### Saccharomycotina

Several strains of *Saccharomyces cerevisiae* contain ergosterol as the major sterol [20] (Figure 2). No other dominant sterols are known in this well-circumscribed group.

#### Pezizomycotina

Most members of the Pezizomycotina produce ergosterol as the major sterol; there are, however, exceptions found in several unrelated taxonomic groups. For example, four species of powdery mildews (Erysiphales), each in a different genus, differ from other Pezizomycotina by their production of 24-methyl-cholesta-5,24(28)-dienol (24-methylene cholesterol) [21], [22] (Figure 2; Table S1). No other members of this group have been analyzed. Three ectomycorrhizal pezizalean species in the genera *Tuber* and *Terfezia* have in common with Taphrinales the accumulation of brassicasterol as a major sterol [23]. *Tuber* contains a mixture of ergosterol and brassicasterol (Figure 2; Table S1).

### Basidiomycota

#### Pucciniomycotina

This diverse taxon contains among other organisms a large group of plant parasites, the rust fungi (Pucciniales). The rust fungi contain 24-ethyl-cholesta-7,24(28)-dienol (stigmasta-7,24(28)-dienol) or 24-ethyl-cholest-7-enol (stigmast-7-enol) as major sterols [24]. These sterols are typically intermediates in the formation of 24-ethyl cholesterol. Apparently, unlike higher plants, the final double bond transformations (Δ^7^ > Δ^5,7^ > Δ^5^) to complete the synthesis to the end-product, i.e., Δ^5^, do not occur in the rust fungi, resulting in Δ^7^ and Δ^7,24(28)^ sterols.

#### Ustilaginomycotina

The smut fungi (Ustilaginales) contain ergosterol as the dominant sterol (Fig. 2; Table S1). As with the Pucciniomycotina, the Ustilaginomycotina are much more diverse than the plant pathogenic smut fungi we associate with the group, and additional sampling throughout the entire subphylum is needed in addition to the few smuts sampled previously.

#### Agaricomycotina

Ergosterol is the major sterol in members of the Agaricomycotina, except for three taxa (*Coriolus sanguineus*, *Fomes applanatus*, and *Polyporus pargamenus*) in the core polyporoid clade [25] that produce 5- or -22- dihydroergosterol [26]–[28] (Figure 2; Table S1). As with other groups of basidiomycetes, sterol analyses should be extended to include more of the diversity of the Agariomycotina.

## Discussion

In view of studies of a wider taxonomic spectrum of fungi studied, it is clear that ergosterol cannot be considered the sole “fungal sterol” because there are taxon-specific sterols other than ergosterol that occur throughout the kingdom Fungi. As demonstrated by us, the previous generalization is an over simplification of a more complex history of sterol structural evolution in fungi.

The presence of non-ergosterol sterols in certain later diverging obligately symbiotic groups within clades of ergosterol-containing fungi has raised questions about their origin, i.e., whether of host or symbiont origin, such as the rust fungi and ectomycorrhizal ascomycetes (see above). In the case of the mycorrhizal fungi, d*e novo* synthesis of 24-ethyl-cholesterol by *Glomus intraradices* has been demonstrated using labeling experiments with [1-^14^C] sodium acetate as a precursor in symbiotic, detached, and germinating stages of the fungus [29]. The C-24 ethyl group of sterols in higher plants (sitosterol) has the α-configuration. The configuration of the ethyl group at C-24 in the fungal sterols has not been determined. In the case of rust fungi, it appears that the sterols are of fungal origin since they were isolated from mycelium grown in axenic culture [30].

There is a progressive shift from considerable structural diversity of dominant sterols among fungal taxa to a single dominant sterol, ergosterol. Cholesterol does not appear to be produced by the later diverging fungi, and ergosterol is not found in the earliest diverging lineages (Figure 2; Table S1). In view of this, cholesterol may be considered the most “primitive” sterol, and the evolution of the specific structure of ergosterol was driven by functional requirements specific to evolving fungi.

The formation of ergosterol requires three reactions that are not involved in cholesterol biosynthesis, i.e., 1) methylation at C-24, 2) reduction at C-24(28), and 3) insertion of the double bond in the side-chain at C-22. The ability to alkylate at C-24 appears to have occurred early in fungal evolution since C-24 methyl and ethyl sterols occur in some of the chytrids. It seems plausible that the ability to produce ergosterol evolved through a series of progressive steps corresponding to the biosynthetic steps leading to this sterol, and that the intermediates accumulated as major sterols in evolutionarily intermediate taxa. The dominance of 22-dihydroergosterol and the absence of ergosterol in Kickxellomycotina (DHK clade of zygosporic fungi) supports the hypothesis that intermediates in ergosterol biosynthesis may accumulate in taxa from which ergosterol-producing taxa evolved.

The selection pressures involved in the evolutionary change from cholesterol to ergosterol as a primary component in fungi are not understood. The driving force for the evolution of ergosterol rather than cholesterol is not intuitive for several reasons. More energy is required for ergosterol synthesis than for cholesterol, and in addition cholesterol has been shown to be the most functionally competent structure in artificial membrane systems [31]. Nevertheless, although ergosterol best satisfies the sterol requirement for growth of yeast [32]–[34], structure-function studies with different sterols have failed to show the advantage of the double bond at C-7 (Δ^5,7^ configuration) and C-22 in ergosterol [35].

Multiple functions for sterols have been identified in yeasts [32]–[34] including a bulk membrane role with relatively low structural specificity and other unidentified roles that have high structural specificity for ergosterol but that require less sterol than the bulk membrane role. It may be that the yet to be identified role(s) is the function that drove the evolution of the ergosterol structure rather than the less specific membrane role.

This study has helped to clarify the available information on sterol distribution in the kingdom Fungi by placing it in a modern phylogenetic context and has identified areas for additional sampling (both for sterols and genes), especially of certain fungal clades in the early diverging lineages and core polypore taxa. It will be useful to clarify more fully how sterols are distributed among fungi and sister taxa [36] and to determine if these biochemical characters have additional phylogenetic relevance. It is clear, however, that ergosterol cannot be reliably used as a marker for all fungal biomass.

## Supporting Information

Table S1 Species sampled from the literature and their major sterols listed in phylogenetic arrangement.
(0.09 MB DOC)
