# Supplementary material for: Phylogenetic Distribution of Fungal Sterols
Source: PLoS One. 2010 May 28;5(5):e10899. doi: 10.1371/journal.pone.0010899 (PMC2878339; doi:10.1371/journal.pone.0010899)
Supplement: Table S1 — Species sampled from the literature and their major sterols listed in phylogenetic arrangement. (0.09 MB DOC) [file pone.0010899.s001.doc]

**Table 1.** Species sampled from the literature and their major sterols listed in phylogenetic arrangement.

| **Phylum Chytridiomycota** |
| --- |
| **Order Chytridiomycetales** |
| *Chytridium confervae* – Cholesterol |
| *Hypochytrium catenoides* – Cholesterol |
| *Rhizophlyctis rosea* – Cholesterol |
| *Rhizophydium sphaerotheca* – 24-Methylene-cholesterol |
| *Zygorhizidium planktonicum* – Cholesterol |
| **Order Spizellomycetales** |
| *Spizellomyces punctatum* – 24-Methyl-cholesterol |
| **Order Monoblepharidales** |
| *Monoblepharella* sp. – 24-Ethyl-cholesterol |
| **Phylum Blastocladiomycota** |
| *Allomyces macrogynus* – Cholesterol |
| *Blastocladiella emersonii* – Cholesterol |
| *Catenaria anguillulae* – Lanosterol |
| **Zygosporic fungi** |
| **Order Mucorales** |
| *Absidia corymbifera* – Ergosterol |
| *Absidia glauca* – 22-Dihydroergosterol |
| *Absidia spinosa* – Ergosterol/22-Dihydroergosterol |
| *Benjaminella youngii* – Ergosterol/22-Dihydroergosterol |
| *Circinella angarensis* – Ergosterol |
| *Cunninghamella echinulata* – Ergosterol |
| *Cunninghamella homothallicus* – Ergosterol |
| *Chaetocladium brefeldii* – Ergosterol |
| *Choanephora cucurbitarum* – Ergosterol/Episterol |
| *Dichotomocladium hesseltinei* – Ergosterol/Episterol |
| *Dichotomocladium floridanum* – Ergosterol |
| *Ellisomyces anomalus* – Ergosterol |
| *Gilbertella persicaria* – Ergosterol |
| *Helicostylum pulchum* – Ergosterol |
| *Hesseltinella vesiculosa* – Ergosterol |
| *Kirkomyces cordensis* – Ergosterol |
| *Micromucor ramanniana* – Ergosterol |
| *Mucor* 7 spp. – Ergosterol |
| *Mucor pusillus* – Ergosterol/22-Dihydroergosterol |
| *Mycotypha microspora* – Ergosterol |
| *Syzygites megalocarpus* – Ergosterol/Episterol |
| *Umbellopsis nana* – Ergosterol |
| *Phycomyces blakesleeanus* – Ergosterol/Episterol |
| *Pilaria anomala* – Ergosterol |
| *Radiomyces mexicanus* – Ergosterol |
| *Rhizopus arrhizus* – Ergosterol |
| *Rhizopus stolonifer* – Ergosterol |
| *Uthraromyces epallocaulus* – Ergosterol/Episterol |
| *Zygorhynchus heterogamus* – Ergosterol |
| **Order Mortierellales** |
| *Lobosporangium transversalis* – Desmosterol |
| *Mortierella alpina* – Desmosterol/24-Methylene-cholesterol |
| *Mortierella chlamydospora* – 24-Methylene-cholesterol |
| *Mortierella elasson* – 24,25-Methylene-cholesterol |
| *Mortierella multidivaricata* – Desmosterol |
| *Mortierella polycephala* – Desmosterol |
| *Mortierella stylospora* – 24,25-Methylene-cholesterol |
| *Mortierella verticillata* – Desmosterol |
| **Order Kickxellales** |
| *Coemansia erectus* – 22-Dihydroergosterol |
| *Linderina pennispora* – Fungisterol |
| *Dipsacomyces acuminosporus* – 22-Dihydroergosterol |
| *Martensiomyces pterosporus* – 22-Dihydroergosterol |
| *Spiromyces aspiralis* – 22-Dihydroergosterol |
| **Order Dimargaritales** |
| *Dimargaris bacillospora* – Ergosterol |
| *Dispira simplex* – Ergosterol |
| *Tieghemiomyces californicus* – Ergosterol |
| **Order Zoopagales** |
| *Syncephalis spherica* – Ergosterol |
| *Zoophagus insidians* – Ergosterol |
| **Order Entomophthorales** |
| *Conidiobolus adieretus* – 24-Methyl-cholesterol |
| *Conidiobolus bangalorensis* – 24-Methyl-cholesterol |
| *Conidiobolus obscura* – 24-Methyl-cholesterol |
| *Conidiobolus virulenta* – 24-Methyl-cholesterol |
| *Delacroixia coronatus* – Cholesterol |
| *Entomophthora destruens* – 24-Methyl-cholesterol |
| *Entomophthora gigantea* – 24-Methyl-cholesterol |
| *Entomophthora pyriformis* – 24-Methyl-cholesterol |
| **Family Basidiobolaceae** |
| *Basidiobolus ranarum* – 24-Methyl-cholesterol/24-methylene-cholesterol |
| **Phylum Glomeromycota** |
| *Acaulospora laevis* – 24-Ethyl–cholesterol |
| *Acaulospora scrobiculata* – 24-Ethyl–cholesterol |
| *Gigaspora margarita* – 24-Ethyl–cholesterol |
| *Glomus* 14 spp. – 24-Ethyl–cholesterol |
| **Phylum Ascomycota** |
| **Subphylum Taphrinomycotina** |
| *Pneumocystis carinii –* Cholesterol |
| *Taphrina* 26 spp. *–* Brassicasterol |
| *Protomyces* 4 spp. – Brassicasterol  *Schizosaccharomyces pombe* – Ergosterol |
| **Subphylum Saccharomycotina** |
| *Saccharomyces cerevisiae* and others – Ergosterol |
| **Subphylum Pezizomycotina** |
| *Alternaria* 4 spp. – Ergosterol |
| *Aspergillus* 4 spp. – Ergosterol |
| *Aureobasidium pullulans* – Ergosterol |
| *Cenococcum geoforme* – Ergosterol |
| *Cercospora arachidicola* – Ergosterol |
| *Cladosporium* sp. – Ergosterol |
| *Cladosporium sphaerospermum* – Ergosterol |
| *Claviceps* sp. – Ergosterol |
| *Erysiphe graminis* – Ergosta-5,24(28)-dienol |
| *Fusarium roseum* – Ergosterol |
| *Hortea werneckii* – Ergosterol |
| *Hypomyces chlorinus* – Ergosterol |
| *Mycosphaerella berkeleyi* – Ergosterol |
| *Neurospora crassa* – Ergosterol |
| *Penicillium* 5 spp. – Ergosterol |
| *Podosphaera leutotrichia* – Ergosta-5,24(28)-dienol |
| *Sphaerotheca fuliginea* – Ergosta-5,24(28)-dienol |
| *Spicaria elegans* – Ergosterol |
| *Terfezia sp.* – Brassicasterol/Ergosterol |
| *Tuber melanosporum* – Brassicasterol/Ergosterol |
| *Tuber brumale* – Brassicosterol/Ergosterol |
| *Uncinula necator* – Ergosta-5,24(28)-dienol |
| **Phylum Basidiomycota** |
| **Subphylum Pucciniomycotina** |
| *Cronartium fusiforme* – Stigmast-7-enol |
| *Gymnosporangium claviceps* – Stigmasta-7,24(28)-dienol |
| *Gymnosporangium juniperi-virginiae* – Stigmasta-7,24(28)-dienol |
| *Puccinia graminis* – Stigmast-7-enol |
| *Puccinia striiformis* – Stigmast-7-enol |
| *Melampsora lini* – Stigmast-7-enol |
| *Uromyces phaseoli* – Stigmasta-7,24(28)-dienol |
| **Subphylum Ustilaginomycotina** |
| *Ustilago maydis* – Ergosterol |
| *Ustilago nuda* – Ergosterol |
| **Subphylum Agaricomycotina** |
| *Agaricus bisporus* – Ergosterol |
| *Agaricus campestrls –* Ergosterol |
| *Calvatia gigantea* – Ergosterol |
| *Clitocybe illudens* – Ergosterol |
| *Daedalea quercina* – Ergosterol |
| *Lenzites trabea* – Ergosterol |
| *Tricholoma rudum* – Ergosterol |
| *Paxillus involutus* – Ergosterol |
| *Scleroderma citrinum* – Ergosterol |
| *Suillus bovinus* – Ergosterol |
| *Suillus variegatus* – Ergosterol |
| *Coriolus sanguineus* – 5-Dihydroergosterol |
| *Fomes applanatus –* 22-Dihydroergosterol |
| *Polyporus pargamenus* *–* 22-Dihydroergosterol |

Names of higher taxa are shown in bold typeface. Note that some taxa are poorly sampled.
